# The Rim and the Ancient Mariner: The Nautical Horizon Affects Postural Sway in Older Adults

**DOI:** 10.1371/journal.pone.0166900

**Published:** 2016-12-14

**Authors:** Justin Munafo, Michael G. Wade, Nick Stergiou, Thomas A. Stoffregen

**Affiliations:** 1 School of Kinesiology, University of Minnesota, Minneapolis, Minnesota, United States of America; 2 Department of Biomechanics and the Center for Research in Human Movement Variability, University of Nebraska-Omaha, Omaha, Nebraska, United States of America; University of California Merced, UNITED STATES

## Abstract

On land, the spatial magnitude of postural sway (i.e., the amount of sway) tends to be greater when participants look at the horizon than when they look at nearby targets. By contrast, on ships at sea, the spatial magnitude of postural sway in young adults has been greater when looking at nearby targets and less when looking at the horizon. Healthy aging is associated with changes in the movement patterns of the standing body sway, and these changes typically are interpreted in terms of age-related declines in the ability to control posture. To further elucidate the mechanisms associated with these changes we investigated control of posture in a setting that poses substantial postural challenges; standing on a ship at sea. In particular, we explored postural sway on a ship at sea when older adults looked at the horizon or at nearby targets. We evaluated the kinematics of the center of pressure in terms of spatial magnitude (i.e., the amount of sway) and multifractality (a measure of temporal dynamics). We found that looking at the horizon significantly affected the multifractality of standing body, but did not systematically influence the spatial magnitude of sway. We discuss the results in terms of age-related changes in the perception and control of dynamic body orientation.

## Introduction

Healthy aging is associated with an increase in the spatial magnitude of standing body sway [1–3]. In plain terms, older adults tend to sway more than younger adults. Traditionally, this age-related increase in sway has been interpreted as a decrease in the stability of postural control [4]. In part, this interpretation is based on the fact that aging also is associated with an increased risk of falling. Many researchers have assumed that the age-related increase in falling is caused, in part, by the age-related increase in body sway [5]. This logical link appears to be simple and straightforward, but it is complicated by two facts. First, healthy older adults are able to modulate the amount of their own body sway. Second, the *amount* of sway differs qualitatively from the temporal structure of sway [2]. These facts raise questions about the traditional interpretation of age-related increases in the spatial magnitude of body sway as an overall decrease in the stability of postural control [6–8]. In the present study, we evaluated both the spatial magnitude and the temporal dynamics of postural sway in healthy older adults. We did this on a ship at sea, a setting that powerfully influences the control of posture in younger adults. Our results provide novel and naturalistic support for new interpretations of postural control and postural stability in older adults.

### Task-related modulation of sway

Standing upright is rarely the sole activity in which a person engages. While standing, we look, listen, manipulate objects, or simply think. The goal of postural control is to maintain the body’s center of mass over the base of support (the feet), but the goals of non-postural tasks often differ qualitatively. For example, if we read while standing, the success of reading is defined in terms of reading rate or comprehension, which differs qualitatively from the position or motion of the center of mass. Activities that co-occur with stance but have different goals are referred to as *supra-postural tasks* [9]. Extensive research has made it clear that properties of supra-postural tasks routinely influence the amount of sway [4]. As one example, postural sway during reading typically has reduced the amount of sway, relative to sway when looking at a blank target [9–10]

As another example, in healthy young adults the spatial magnitude of sway is greater when looking at more distant visual targets than when looking at nearby visual targets [11–13]. This same effect occurs among healthy older adults, despite the fact that the overall magnitude of sway was greater for older adults than for younger adults [3]. Effects of supra-postural tasks suggest that healthy older adults retain the ability to modulate their postural sway, and are not readily compatible with the traditional idea that the increased postural sway of older adults represents uncontrolled instability [7–8]. In the present study, we varied the distance of visual targets and evaluated the effects of this manipulation on the standing body sway of healthy older adults in a novel setting; a ship at sea.

### Postural control on a moving surface

Assessments of postural activity often focus on situations in which there were no external perturbations to posture, sometimes referred to as the *quiet stance paradigm* [4]. Posture must also be controlled in the presence of perturbations, that is, sources of motion outside the body, often referred to as the *perturbation paradigm* [14]. In the laboratory, perturbations to posture often take the form of motion of the surface upon which the person is standing [15]. This paradigm has been very productive, but it has fundamental limitations. Among these are limits on the magnitude and complexity of perturbations that can be applied. Laboratory devices typically can move less than 1 m, with motion that is brief (1 s or less), and limited to a single axis. Outside the laboratory it is possible to identify situations that feature larger, more complex, and more persistent displacements. Postural activity in the presence of complex, long-duration perturbations can have implications for general theories of perceptual-motor control [16–17].

**Fig 1:**
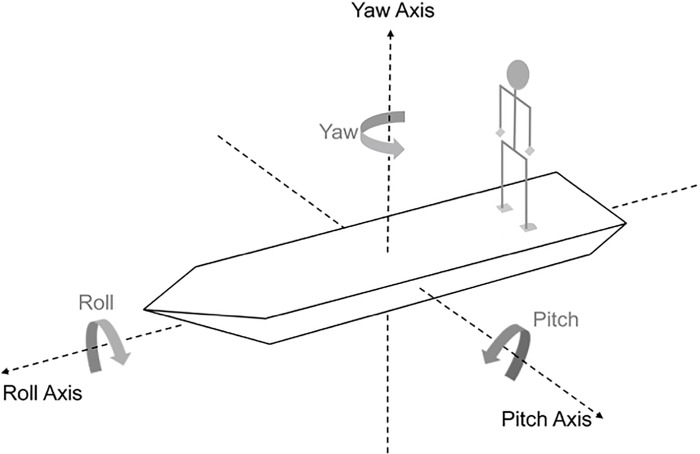
Illustration of ship motion, showing roll, pitch, and yaw, the three rotational degrees of freedom. The three translational degrees of freedom are surge, sway, and heave, which are motions along the roll, pitch, and yaw axes, respectively.

One example of non-laboratory perturbation that has proved generative for research on postural control is the motion of ships at sea. Motion of the surface of the sea (i.e., waves and swell) displaces ships, leading to oscillatory ship motion in 6 degrees of freedom (Fig 1), which commonly is on the order of meters, and which persists across hours and days. Ship motion is a powerful constraint on control of the body, a fact that has been known anecdotally for millennia [18]. Experimental research has confirmed anecdotal accounts, and has provided insight into quantitative details about how postural control can be adapted to life on moving surfaces.

We can evaluate the impact of ship motion on postural control by considering measures of effect size relating to variations in ship motion. One common measure of effect size is partial η^2^, which ranges from 0.0 to 1.0. Cohen [19] suggested that values of partial η^2^ greater than 0.26 are “large.” Variations in ship motion have yielded statistically significant effect sizes ranging from 0.60 to 0.99 [20–24], which are exceptional within the behavioral sciences, and which testify to the powerful effects of ship motion on the control of the body.

#### The role of the horizon: Land versus sea

As noted above, the spatial magnitude of postural activity is related to the egocentric distance of visible targets. The logical limit of this relationship occurs when people look at the horizon. On land, the spatial magnitude of postural sway is greater when looking at the visible horizon than when looking at nearby targets, in both younger and older adults [11, 16, 25]. Typically, these effects are stronger for postural sway in the body’s anterior-posterior (AP) axis than for sway in the body’s mediolateral (ML) axis. The influence of the visible horizon on the spatial magnitude of postural sway has been referred to as the *Grand Canyon effect* [21].

The most common interpretation of the Grand Canyon effect relies on the projective geometry of the optical consequences of body sway in relation to the visible surroundings [12, 26–28]. A given amplitude of body sway creates greater change in optic flow arising from nearby objects and surfaces, and less change in optic flow arising from distant objects and surfaces. For objects and surfaces at very large distances, body sway should yield optical changes that are too small to be detected by the visual system. Accordingly, nearby visual targets should play a greater role than the horizon in the control of body posture. This analysis appears to imply that viewing of the horizon should not stabilize body posture at sea. Specifically, this view would predict that, at sea, posture should be more stable when looking at the ship than when looking at the horizon.

The terrestrial Grand Canyon effect contrasts with phenomena that are associated with sea travel. Stretching back over several millennia, anecdotal reports suggest that standing on the open deck and looking at the nautical horizon (the rim of the world) can improve both subjective stability and the control of body sway. That is, anecdotal evidence suggests that the effect of the visible horizon at sea is exactly the opposite of what it is on land. Recent experiments have confirmed the anecdotal reports. On ships at sea, when standing on the open deck the spatial magnitude of standing body sway is *reduced* when participants look at the horizon, relative to sway when looking at nearby targets on the ship. This effect has been observed among experienced maritime crewmembers [16], and in young adult maritime novices [21]. These nautical effects raise questions about the traditional, geometrical interpretation. In the present study, we asked whether the nautical horizon would affect the control of standing body sway among healthy older adults on a ship at sea.

### Multifractality in postural sway

The interpretation of movement variability has been profoundly affected by developments in dynamic systems theory, with special emphasis on the temporal properties of behavior. One consequence of these developments is growing debate about the definition of *stability* in the context of human movement, in general, and postural control, in particular [2, 7, 29]. Postural sway having a given spatial magnitude can be more stable or less stable, depending on its temporal properties. In general, the spatial and temporal structure of movement differ qualitatively, such that one cannot be reduced to (or deduced from) the other. For this reason, in the present study, we separately assessed the spatial magnitude and the temporal dynamics of postural sway. We evaluated the spatial magnitude of sway in terms of the positional variability, a widely used measure [9–10, 13], which is similar to the root mean square of position [3, 30]. We evaluated temporal dynamics in terms of the multifractality of postural sway.

In early work, it was assumed that the degree of fractality was constant, for a given individual. More recent research has demonstrated that the nature of fractality can change. Changes in fractality demonstrate multifractality. Multifractality has been identified in both cognitive [31] and perceptual-motor processes [32–34]. Some scholars have suggested that multifractaility is a fundamental aspect of animate behavior, in general [31], and of perceptual-motor behavior, in particular [32, 35]. That is, multifractality should be an intrinsic property of the system and, in this sense, may differ from other aspects of temporal dynamics. Several scholars have argued that measures of multifractality may be more meaningful than measures of other aspects of temporal dynamics [31, 33, 36]. In this article, we do not recapitulate these arguments; rather, we use them to motivate the use of multifractality as a measure of the temporal dynamics of body sway in our study.

Several studies have documented the existence of multifractality in standing body sway [32, 34, 37]. Ihlen et al. [32] and Goldberger et al. [38] suggested that age-related changes in physiology and neurology might lead to a reduction in the multifractality of movement. Munafo et al. [25] compared the multifractality of standing sway in younger and older healthy adults. They confirmed the existence of an age effect; however, the degree of multifractality was *greater* in older adults than in younger adults, directly contrary to the prediction of Goldberger et al.

Munafo et al. [25] also investigated the influence of the horizon on postural sway in younger and older adults. In addition to evaluating the spatial magnitude of postural sway, they separately evaluated the multifractality of sway. They found a statistically significant interaction between target distance and age groups, revealing that older adults systematically modulated the width of the multifractal spectrum as a function of the distance of visual targets, while younger adults did not. That is, among older adults, multifractality scaled negatively with target distance; that is, multifractality was greater for nearby targets, and was reduced for the horizon. This effect was the first empirical demonstration that the distance of visual targets can affect the multifractality of postural sway. However, the nature of the effect (that target distance affected the amount of sway of older adults but not younger adults) does not appear to be compatible with the traditional assumption that the stability of postural control declines with age.

### The present study

In the present study, we asked whether the nautical horizon would affect the spatial magnitude of standing body sway in older adults in the same way that it affects standing body sway in younger adults [21]. In older adults, we predicted that the spatial magnitude of postural sway would be reduced when looking at the nautical horizon, relative to sway when looking at a nearby target. Second, we asked whether the nautical horizon would affect the multifractality of standing body sway in older adults. By analogy with previous nautical research [16, 21], in which the effect of the horizon on land differed qualitatively from the effect of the horizon at sea, we predicted that multifractality would be greater when looking at the nautical horizon than when looking at a nearby target. Finally, following previous studies, we predicted that effects of the horizon would be greater for sway in the body’s AP axis than for sway in the body’s ML axis.

## Materials and Methods

We sought to maximize the extent to which our study of older maritime novices would be comparable to previous studies of younger adult maritime novices. For this reason, the present study was conducted in the same location on the same ship as the study of Stoffregen et al.] 21], departing from the same port in the same season and under similar sea conditions. With respect to our variation in the distance of visual targets, we used a within-participants design. With respect to our variation in testing days, our design was between-participants.

### Ethics Statement

The experimental protocol was approved in advance by the University of Minnesota IRB, and informed consent was obtained from each participant in writing.

### Participants

Participants were 18 adults (11 males, 7 females) ranging in age from 56 to 78 years (M = 66.16 years), who were paying passengers on an Enrichment Voyage operated by the Institute for Shipboard Education (www.semesteratsea.com). We did not formally assess health status. Participants were healthy in the sense that they were bi-pedally ambulatory (i.e., they walked without support or assistance), were sufficiently independent that they had elected to embark upon a sea voyage, and were willing and able to stand on the open deck for the duration of our protocol. On Day 1, there were 6 men and 5 women and the age range was 56–72 years, with a mean of 65.1 years. On Day 2, there were 5 men and 2 women and the age range was 56–78 years, with a mean of 67.3 years.

### Apparatus and experimental setting

The study was conducted on the *M/V Explorer*, which was 86 m long, with a 16 m beam and displacing 25,000 tons. During testing, the ship travelled at approximately 13 knots. The experiments were conducted on the aft end of deck 4, an open space approximately 20 m wide by 10 m deep. This was the same space used by Stoffregen et al. [21]. A safety railing surrounded the perimeter; otherwise, the area provided an unimpeded view of the ocean from the ship’s stern.

We evaluated postural activity during stance on a force plate (Accusway, AMTI, Watertown, MA), which was sampled at 50 Hz in the AP and ML axes. This device was one of two force plates used by Stoffregen et al. [21].

### Procedure

**Fig 2:**
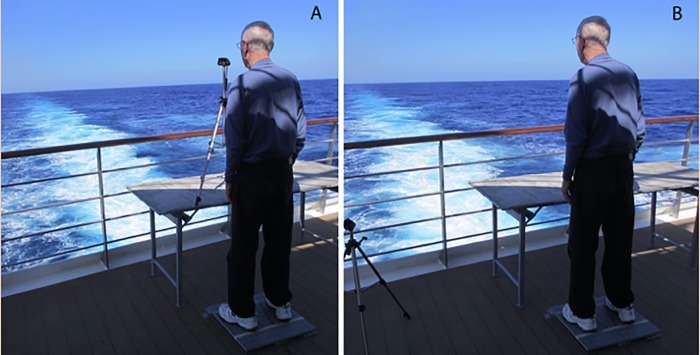
Experimental setting. A. Near target condition. B. Far target (horizon) condition.

The ship departed Nassau, The Bahamas, on the evening of December 18 2013 for a 3-week cruise. We collected data during the first two full days at sea (December 19 and 20). The experimental setting is illustrated in Fig 2. Data were collected in the same physical location on the same ship, in the same waters, in the same season, and using the same force plate as in Stoffregen et al. [21].

Participants were tested while wearing their shoes. The participants wore loafers or athletic shoes. We did not recruit individuals who were wearing high heels. The procedure was the same as Experiment 2 from Stoffregen et al. [21]. Participants stood on marked lines on the force place such that there were 17 inches between heels and the feet were at an angle of 10° relative to each other. In the near target condition participants were asked to keep their gaze on a tripod head, located 50 cm in front of the participant’s heel. In the far target condition, the tripod was removed and the participant was instructed to look straight ahead at the horizon. Individual trials were 60 s in duration. Each participant completed three trials in the near target condition and three in the far target condition, with condition order counter-balanced across participants. Each day, data were collected from 9:00–11:30 and 13:00–16:00.

### Data analysis

We evaluated the amount, or spatial magnitude of body sway, and we separately evaluated the temporal dynamics of body sway. The spatial magnitude of body sway can be assessed in many ways, including its range, area [2]. To enable direct comparison with certain previous studies, we evaluated the spatial magnitude of sway in terms of the positional variability of the center of pressure, which we defined operationally as the standard deviation of center of pressure positions [9, 13, 16, 21]. For each trial, we computed the standard deviation of the center of pressure time series. These standard deviations were the data used in our inferential statistics. Thus, the means that we report for positional variability are the means (across conditions) of these per-trial standard deviations.

We evaluated the multifractality of sway in terms of multifractal detrended fluctuation analysis, or MF-DFA. MF-DFA is an extension of detrended fluctuation analysis [2], which provides access to a qualitatively different type of information. Traditional DFA yields α, the scaling exponent, but assumes homogenous fluctuations in a time series [31]. For this reason, traditional DFA cannot be used to assess the existence or degree of multifractality in time series data. Multifractal fluctuations are interdependent and heterogeneous. The range of the singularity exponent, *h*(*q*), can be used as an index of the degree of multifractality in a time series [39–40]. The range of *h(q)* values is known as the *singularity spectrum*, or simply the *spectrum*. The wider the multifractal spectrum, the more multifractal is the movement [36]. For each trial, we computed *h(q)* using open source code for MATLAB (MFDFA1[39]), set to a minimum scale of *q* = −10, increasing incrementally by 1 to *q* = 10, and a polynomial trend fit set to 3. We selected a minimum scaling range of 16 data points with 19 evenly spaced increasing segment sizes to a maximum of the length of the time series. Each time series comprised 3000 data points.

MF-DFA is a novel technique, particularly in relation to the analysis of human movement. Researchers have argued that multifractality may be an inherent component of animate movement, including but not limited to the kinematics of postural sway [31, 33, 36]. Yet the same researchers have acknowledged that, due to its novelty, the interpretation of data on multifractality often is not straightforward. In the present study, our use of and predictions about MF-DFA were based on published derivations of the analytic technique, and on published empirical studies showing that MF-DFA can be influenced by a variety of behaviorally relevant independent variables [25, 32–34, 37].

For each dependent variable, we conducted separate 3-factor ANOVAs on target distance (Near vs. Far), body axis (AP vs. ML), and days (Day 1 vs. Day 2). Target distance and body axis were within-participants factors, while days was a between-participants factor. We report only effects that reached statistical significance. Munafo et al. [25] directly evaluated relations between the two dependent variables, and found them to be orthogonal to each other. For this reason, in the present study we did not deem it necessary to conduct a multivariate ANOVA to control for a relationship between our dependent variables.

## Results

### Ship motion

The weather was partly cloudy with occasional squalls of rain. We estimated sea state using the Beaufort scale [41], on which 1 = flat calm, and 10 = hurricane. On Day 1, the sea state was approximately 3.5 on the Beaufort scale. On Day 2, the sea state gradually increased from 2 to 3. For comparison, in Experiment 2 from Stoffregen et al. [21], the sea state was 4 on the first day of testing, and 5 on the second day. Variations in sea state can affect the kinematics of standing body sway [42]. Accordingly, the conditions in the present study were not identical to those of Stoffregen et al. [21]. For post-hoc tests from statistically significant interactions, the criterion alpha was set at .005.

### Postural sway

**Fig 3:**
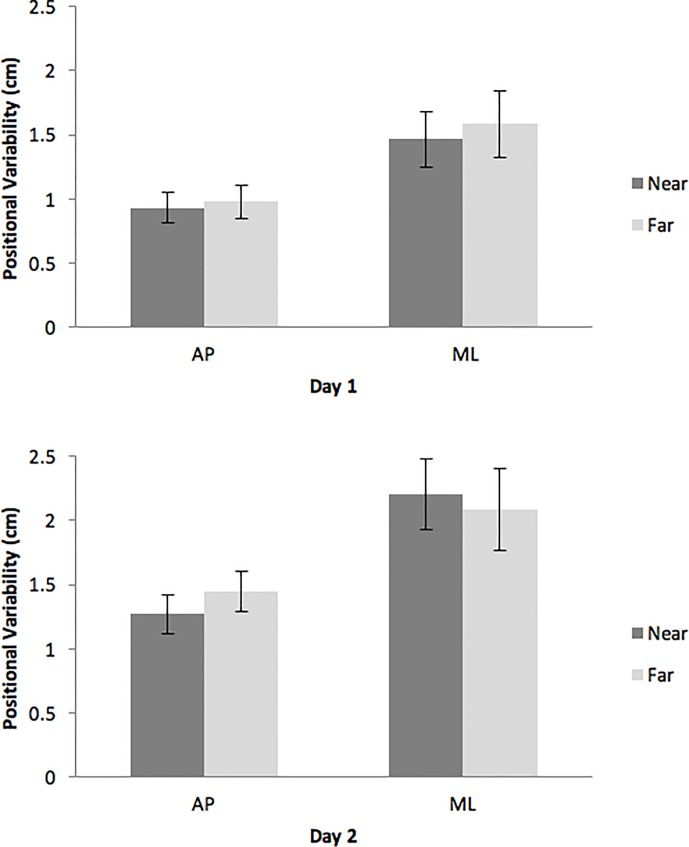
Positional variability of the center of pressure, illustrating the statistically significant condition × body axis × days interaction. The error bars represent the 95% confidence interval of the mean.

In our analysis of the positional variability of the center of pressure, the main effect of body axis was significant, F(1,16) = 59.66, *p* < .01, partial η^2^ = 0.79. Positional variability was greater in the body’s ML axis (mean = 1.83 cm, SD = 0.09 cm) than in its AP axis (mean = 1.12 cm, SD = 0.04 cm). The main effect of days was significant, F(1,16) = 23.48, *p* < .01, partial η^2^ = 0.60. Positional variability was greater on Day 2 (mean = 1.75 cm, SD = 0.08 cm) than on Day 1 (mean = 1.24 cm, SD = 0.07 cm). In addition, the condition × body axis × days interaction was significant, F(1,16) = 9.23, *p* < .01, partial η^2^ = 0.37 (Fig 3). Post-hoc tests revealed only one significant contrast involving target distance: On Day 2, positional variability in the AP axis was greater during viewing of the horizon than during viewing of the nearby target, *t*(6) = 5.69, *p* = .0013.

**Fig 4:**
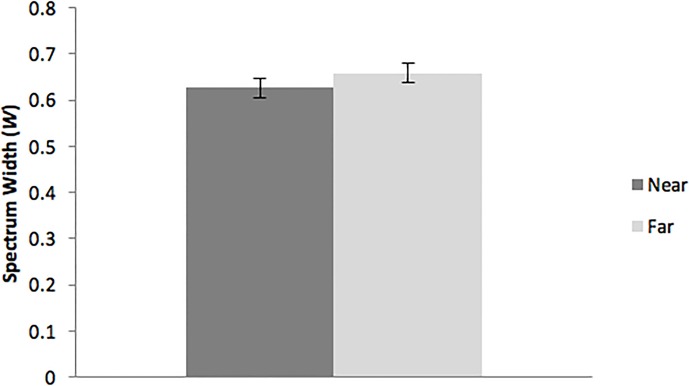
The width, *W*, of the multifractal spectrum, illustrating the statistically significant effect of target distance. The error bars represent the 95% confidence interval of the mean.

**Fig 5:**
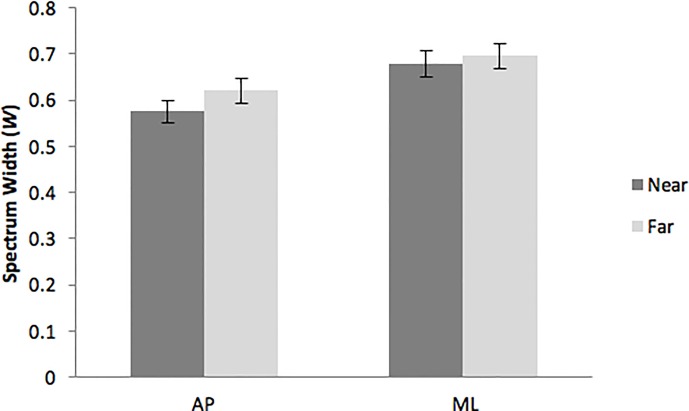
The width, *W*, of the multifractal spectrum, illustrating the statistically significant interaction between conditions and body axis. The error bars represent the 95% confidence interval of the mean.

For the width of the multifractal spectrum, the main effect of axis was significant, F(1,16) = 47.20, *p* < .01, partial η^2^ = 0.75. Spectrum width was greater for sway in the body’s ML axis (mean = 0.69, SD = 0.01), than for sway in the body’s AP axis (mean = 0.60, SD = 0.01). In addition, the main effect of conditions was significant, F(1,16) = 10.54, *p* = .01, partial η^2^ = 0.40. Spectrum width was greater when looking at the horizon than when looking at the nearby target (Fig 4). These main effects were modulated by a significant conditions × axis interaction, F(1,16) = 6.61, *p* = .02, partial η^2^ = 0.29 (Fig 5). Post-hoc tests of this interaction revealed that several significant contrasts. Our variation in target distance affected spectrum width for postural activity in the AP axis, *t*(17) = 4.64, *p* < .001, but not in the ML axis, *t*(17) = 1.25, *p* = .11. When looking at the horizon, spectrum width was greater in the ML axis than in the AP axis, *t*(17) = 5.14, *p* < .001. Also, spectrum width in the ML axis was greater than in the AP axis when viewing the nearby target, *t*(17) = 8.700020043 *p* < .001. Finally, during viewing of the nearby target, spectrum width was greater in the ML axis than in the AP axis, *t*(17) = 8.64, *p* < .001.

### Comparisons between studies

To evaluate effects of age on postural sway at sea, we compared data from the present study with data from Days 1 and 2 from Stoffregen et al. [21], which was conducted in the same location on the same ship at the same season in the same waters under similar sea conditions. As noted earlier, these comparisons are of limited value, given that there were differences in sea states between the two studies. For positional variability of the center of pressure the Grand Mean in the present study (1.50 cm, SD = 0.21 cm) did not differ from the mean reported by Stoffregen et al. (1.66 cm, SD = 0.11 cm), *t*(40) = 0.73, *p* = .24.

To evaluate the effects of ship motion on postural sway in older adults, we compared the Grand Mean of positional variability in the present study versus the mean of the Near and Far conditions for older adults from Munafo et al. [25], who were tested in a terrestrial outdoor setting. The Grand Mean of the present study (1.50 cm, SD = 0.21 cm) was greater than the mean for older adults in Munafo et al. [25] (0.43 cm, SD = .21 cm), *t*(30) = 4.58, *p* < .001, consistent with the finding that the spatial magnitude of body sway is greater at sea than on land [21].

To address effects of ship motion on the multifractality of sway among older adults, we compared the Grand Mean of spectrum width in the present study (0.64, SD = .04) versus the mean for older adults from Munafo et al. [25] (0.51, SD = .06). These means differed significantly, *t*(30) = 7.72, *p* < .001, confirming that effects of ship motion on postural control extend to the multifractality of sway.

## Discussion

On a ship at sea, healthy older adults looked at the horizon, or at a nearby target on the ship. Looking at the horizon influenced the spatial magnitude and the multifractality of postural sway. However, the nature of these effects differed from previous studies of younger adults at sea, and from previous studies of older adults on land.

### Effects of the horizon

The horizon had relatively little effect on the spatial magnitude of sway, as reflected in the positional variability of the center of pressure. The absence of a significant main effect of conditions contrasts with younger adults in a previous study on the same ship [21]. The statistically significant 3-way interaction between conditions (near target vs. horizon), body axis, and days (Fig 3) confirms that the horizon did not have a consistent effect on positional variability. Indeed, post-hoc tests revealed a significant difference between looking at the near target and looking at the horizon only on Day 2, and only for positional variability in the AP axis; and in that case the direction of the effect was opposite to what has been observed in previous research at sea [16, 21]. These results suggest that, in terms of the spatial magnitude of postural activity the effects of the nautical horizon differ markedly between younger and older adults.

In the present study, as in the study of Stoffregen et al. [21], data were collected during the first two days of the voyage, that is, during the period in which passengers were getting their sea legs. It may be that the process of adapting to ship motion (that is, the process of getting one’s sea legs) may take longer in older adults than in younger adults. Age-related delays in perceptual-motor learning have been documented in some contexts [43–44], but not in others [45]. It might be that the relatively weak effects of the nautical horizon on the positional variability of older adults’ postural sway were related to a more protracted process of getting one’s sea legs. This hypothesis could be tested by monitoring the postural sway of older adults over longer periods at sea; perhaps a week.

In contrast to the spatial magnitude of postural activity, we found that the nautical horizon had robust effects on the multifractality of sway, as reflected in the width of the multifractal spectrum. The more distant visual target (the horizon) was associated with increased spectrum width, relative to the nearby target (Fig 4). This pattern of results differs qualitatively from effects observed on land, where more distant visual targets were associated with *reduced* spectrum width, relative to nearby targets [25]. The effect of target distance was concentrated in the body’s AP axis (Fig 5), a finding that is consistent with both terrestrial and nautical research [16, 21, 25]. Taken together, the results indicate that the visible horizon primarily affects postural activity in the body’s AP axis, but that the nature of these effects differs qualitative between nautical and terrestrial settings.

With respect to the qualitative difference in the effects of the horizon on postural sway at sea versus on land, our older adult participants resembled younger adults [16, 21]. We conclude that the nautical horizon is an important factor in the control of standing body posture on ships in both younger and older adults. In future research, it will be interesting to consider the role of the horizon in development. Many vacation cruise companies cater to families with children, even to the extent of providing daycare for infants. On such ships it would be possible to evaluate the role of the horizon in the control of body posture for infants, children, and adolescents.

### Effects of age

As noted above, our study was conducted on the same ship, and in the same waters, as Stoffregen et al. [21], who studied younger adults. In addition, the sea state reported by Stoffregen et al. [21] (4 on Day 1, and 5 on Day 2) was similar to that in the present study. Accordingly, it is reasonable to compare the present results, obtained with older adult maritime novices, with postural sway in younger adult maritime novices on Days 1 and 2 [21]. The Grand Mean of positional variability in the present study did not differ from the mean reported for Days 1 and 2 by Stoffregen et al. [21]. This nautical finding contrasts with postural control on land, where many studies have shown that the spatial magnitude of postural sway is greater among older adults than among younger adults [3]. The results of the present study, together with those of other nautical studies [20, 23–24] suggest that ship motion may be such a powerful constraint on control of the body that it can eliminate age-related variations in the spatial magnitude of postural sway. This finding must be regarded as tentative, due to our between-voyages comparison, yet the possibility motivates future research in which younger and older adults are compared, using within-participants designs, both before and during a sea voyage.

Stoffregen et al. [21] found that younger adult maritime novices used the nautical horizon to reduce the spatial magnitude of postural sway after only 24 hours at sea. In the present study, our older adult maritime novice participants had not made a similar change after 48 hours at sea. It is possible that older adults do learn to use the nautical horizon to modulate the spatial magnitude of postural sway, but that they do so more slowly than younger adults. This question could be resolved by monitoring the postural sway of older adults over the course of a longer sea voyage.

### Effects of ship motion

In the present study, the positional variability of sway was greater in the ML axis than in the AP axis. This result differs from terrestrial studies using the same stance width, in which positional variability typically is greater in AP than in ML, in both younger and older adults [6, 13, 16, 21, 25, 46]. The terrestrial effect has been interpreted as arising from biomechanical factors [47], but more recent evidence suggests that it may be related to functional demands of supra-postural tasks [20, 48]. The finding that our participants were able to reverse the relationship between sway in the AP and ML axes highlights the adaptability of older adults in the face of ship motion. Because ships typically are longer than they are wide, ship motion in roll tends to be greater than in pitch. When facing the stern (as in the present study), sway in the body’s ML axis will be affected by ship motion in roll, while sway in the body’s AP axis will be affected by ship motion in pitch. Thus, the observed increase in ML sway may have been an adaptive response to the magnitude of roll motion. A similar effect was observed by Munafo et al. [49], who found, among older adults at sea, that performance in the standing Romberg test was better when facing port or starboard (where performance was primarily influenced by ship motion in pitch) than when facing fore-aft (where performance was primarily influenced by ship motion in roll).

### Testing on different days

Postural activity differed between Day 1 and Day 2. Motion of the ship differed between days, and daily variations in ship motion are known to affect postural sway in younger adults [22, 42]. However, in the present study the differences between days that we observed cannot be interpreted as simple effects of daily variations in ship motion. The reason is that we used a between-participants design in which each individual was tested on only one day. The age of our participants did not differ between days. However, the distribution of male and female participants did differ across days. On Day 1 the number of male and female participants was equal, while on Day 2 there were more men than women. Many studies have shown that there exist reliable differences in postural sway between the sexes [50], including among older adults [51–52], but no previous studies have compared the sexes on ships at sea. These issues can be resolved only through future research.

The multifractality of postural activity did not differ between testing days. In positional variability, the effect size for the main effect of days was very large (0.60). Taken together, the results underscore the independence of positional variability and multifractality, that is, the independence of the spatial magnitude versus the temporal dynamics of movement.

### The meaning of multifractality

Due to its qualitative novelty, researchers have acknowledged that the interpretation of multifracality in terms of functional outcomes is not self-evident [33]. The evaluation of multifractality in relation to known outcomes can help scientists to understand the meaning of these metrics. As one example, the postural control of older adults is widely (perhaps universally) understood to be less stable than that of young adults. Thus, age-related variations may make it possible to interpret certain values of multifractality in terms of stability and instability. Yet overall interpretation is likely to be complex. Multifractality varies with age, but it also varies with variations in visual tasks, as demonstrated in Munafo et al. [25], and in the present study. Palatinus et al. [33] found that the multifratality of torso movement varied as a function of “perceptual intent”, that is, whether participants were attempting to detect the whole length or the partial length of an unseen rod that was attached to their shoulders. Finally, Koslucher et al. [53] found, in young adults, that the multifractality of standing body sway differed between participants who proved to be susceptible to visually induced motion sickness and those who did not become sick. Much additional research will be required to identify reliable links between patterns of multifracality in human movement and particular states, situations, conditions, or diagnoses of the individuals involved.

### Aging and the stability of postural control

Healthy aging is associated with an increase in the spatial magnitude of standing body sway [2]. Traditionally, this common finding has been interpreted as indicating an age-related decline in the ability to stabilize the body [4, 6]. The results of the present study, together with results from other recent research, raise questions about this traditional interpretation. While healthy older adults do sway more, this increase in the spatial magnitude of sway is not involuntary, or inescapable. In a terrestrial laboratory setting, Prado et al. [3] varied the distance of visual targets. Overall postural sway was greater among healthy older adults than among healthy younger adults, replicating the common finding. Yet both age groups modulated the spatial magnitude of sway in response to the variation in the distance of visual targets, and the effects of target distance did not differ between age groups. That is, healthy older adults retained the ability to modulate the spatial magnitude of their postural sway, and their use of this ability did not appear to be affected by age.

The logical limit for the distance of visual targets is the visible horizon. The influence of the visible horizon on postural control has been evaluated in outdoor settings. On land, the spatial magnitude of standing body sway is greater when looking at the visible horizon than when looking at nearby targets for healthy younger adults, and for healthy older adults [25]. These effects are compatible with the hypothesis that healthy older adults retain the ability to modulate postural activity to support the performance of supra-postural tasks [7–8, 54]. To the extent that this hypothesis is correct, then the increased risk of falls among healthy older adults must have a cause other than age-related changes in the control of stance.

## Conclusion

On land, the spatial magnitude of standing body sway is influenced by the distance of visual targets, in both younger and older healthy adults [3]. Among younger adults on ships at sea, the relation between visual target and the spatial magnitude of sway is qualitatively reversed [16, 21]. In the present study, we asked whether the kinematics of standing body sway of healthy older adults would be influenced by the distance of visual targets. On a ship at sea, we measured the kinematics of standing body sway as healthy older adults looked at a nearby visual target, or at the nautical horizon. This variation in visual target distance had no systematic effect on the spatial magnitude of postural sway. However, the distance of visual targets had significant effects on the temporal dynamics of sway, as revealed by our analysis of the width of the multifractal spectrum. The results demonstrate that the nautical horizon, the rim of the world, can influence the kinematics of standing body sway in healthy older adults, but the nature of these effects differs between older and younger adults.

The role of the nautical horizon in the control of stance has special importance among older adults. Ageing in associated with changes in the control of standing body posture and with an increased risk of falling [6, 55]. Therefore, it might be expected that older adults would choose to avoid situations that increase the risk of falls. Motion of ships on the open sea is continuous, complex, multidimensional, and often on the order of several meters. Experimental studies demonstrate that ship motion poses significant challenges for the control of posture. Cohen [19] suggested that values of partial η^2^ greater than 0.26 are “large”. Accordingly, effects in the present study, which ranged in effect size from 0.29 to 0.79, can be interpreted as being substantial, confirming the powerful influence of ship motion on control of the body [20, 23–24], and extending this effect to the domain of healthy older adults. Despite the dramatic influence of ship motion, millions of older persons choose to take sea voyages. Each year, millions of older persons embark upon sea voyages [56], paying large sums of money with the understanding that they will be exposed to unpredictable variations in ship motion. There may be an influence of self-selection, yet the popularity of vacation cruises suggests that, among healthy older adults, ship motion is not viewed as a threat to posture and locomotion. Together with Munafo et al. [49], our study provides the first controlled, experimental demonstrations of how healthy older adults modulate their postural activity to maintain upright stance under the challenging conditions of life at sea.

## References

[1] Kinsella–Shaw J. M., Harrison S. J., Colon–Semenza C., & Turvey M. T. (2006). Effects of visual environment on quiet standing by young and old adults. J Motor Behav 38: 251–264. 10.3200/JMBR.38.4.251-264 16801318

[2] Lin D., Seol H., Nussbaum M. A., & Madigan M. L. (2008). Reliability of COP-based postural sway measures and age-related differences. Gait & Posture, 28, 337–342. 10.1016/j.gaitpost.2008.01.005 18316191

[3] Prado J. M., Stoffregen T. A., & Duarte M. (2007). Postural sway during dual tasks in young and elderly adults. Geront 53: 274–281. 10.1159/000102938 17510558

[4] Woollacott M, & Shumway-Cook A. (2002) Attention and the control of posture and gait: A review of an emerging area of research. Gait Pos 16: 1–14. 10.1016/s0966-6362(01)00156-4 12127181

[5] Merlo A., Zemp D., Zanda E., Rocchi S., Meroni F., Tettamanti M., Recchia A., Lucca U., & Quadri P. (2012). Postural stability and history of falls in cognitively able older adults: The Canton Ticino study. Gait Pos 36: 662–666. 10.1016/j.gaitpost.2012.06.016 22832469

[6] Melzer I., Benjuya N., & Kaplanski J. (2004). Postural stability in the elderly: A comparison between fallers and non-fallers. Age Ageing 33: 602–607. 10.1093/ageing/afh218 15501837

[7] van Emmerik R. E. A., & van Wegen E. E. H. (2002). On the functional aspects of variability in postural control. Exer Sports Sci Revi 30: 177–83. 10.1097/00003677-200210000-00007 12398115

[8] Stoffregen T. A. (2016). Functional control of stance in older adults. Kines Rev 5: 23–29.

[9] Stoffregen T. A., Pagulayan R. J., Bardy B. G., & Hettinger L. J. (2000). Modulating postural control to facilitate visual performance. Hum Move Sci 19: 203–220.

[10] Yu Y., Chung H.-C., Hemingway L., & Stoffregen T. A. (2013). Standing body sway in women with and without morning sickness in pregnancy. Gait Pos 37: 103–107. 10.1016/j.gaitpost.2012.06.021 22824679

[11] Bles W., Kapteyn T.S., Brandt T., & Arnold F. (1980). The mechanism of physiological height vertigo: II. Posturography. Acta Otolaryng 89: 534–540. 6969517 10.3109/00016488009127171

[12] Lee D. N., & Lishman J. R. (1975). Visual proprioceptive control of stance. Journal of Hum Move Stud 1: 87–95.

[13] Stoffregen T. A., Smart L.J., Bardy B.G., & Pagulayan R. J. (1999). Postural stabilization of looking. J Exp Psychol: Hum Percep Perf 25: 1641–1658.

[14] Hirata R., Arendt-Nielsen L., & Graven-Nielsen T (2010). Experimental calf muscle pain attenuates the postural stability during quiet stance and perturbation. Clin Biomech 25: 931–937. 10.1016/j.clinbiomech.2010.06.001 20692746

[15] Nashner L. M., & McCollum G. (1985). The organization of postural movements: a formal basis and experimental synthesis. Behav Brain Sci 26: 135–172.

[16] Mayo A. M., Wade M. G., & Stoffregen T. A. (2011). Postural effects of the horizon on land and at sea. Psychol Sci 22: 118–124. 10.1177/0956797610392927 21156861

[17] Riccio G.E. (1995). Coordination of postural control and vehicular control: Implications for multimodal perception and simulation of self-motion In Hancock P., Flach J., Caird J., & Vicente K. (Eds.), Local applications of the ecological approach to human- machine systems (pp. 122–181). Mahwah, NJ: Erlbaum.

[18] Stevens S. C., & Parsons M. G. (2002). Effects of motion at sea on crew performance: A survey. Marine Tech 39: 29–47.

[19] Cohen J. (1988) Statistical power analysis for the behavioral sciences (2nd ed.). Hillsdale, NJ: Erlbaum.

[20] Chen F.-C., & Stoffregen T. A. (2012). Specificity of postural sway to the demands of a precision task at sea. J Exp Psychol: App 18: 203–212. 10.1037/a0026661 22181030

[21] Stoffregen T. A., Chen F.-C., Varlet M., Alcantara C., & Bardy B. G. (2013). Getting your sea legs. PLOS ONE, 8(6), e66949 10.1371/journal.pone.0066949 23840560 PMC3686767

[22] Stoffregen T. A., Villard S., Chen F.-C., & Yu Y. (2011). Standing body sway on land and at sea. Ecol Psychol 23: 19–36.

[23] Varlet M., Bardy B. G., Chen F.-C., Alcantara C., & Stoffregen T. A. (2015). Coupling of postural activity with motion of a ship at sea. Exp Brain Res 233: 1607–1616. 10.1007/s00221-015-4235-7 25716613

[24] Varlet M., Stoffregen T. A., Chen F.-C., Alcantara C., Marin L., & Bardy B. G. (2014). Just the sight of you: Postural effects of interpersonal visual contact at sea. J Exp Psychol: Hum Percep Perf 40: 2310–2318. 10.1037/a0038197 25314043

[25] Munafo J., Curry C., Wade M. G., & Stoffregen T. A. (2016). The distance of visual targets affects the spatial magnitude and multifractal scaling of standing body sway in younger and older adults. Exp Brain Res 234:2721–2730. 10.1007/s00221-016-4676-7 27255223

[26] Paulus W., Staube A., Krafczyk S., & Brandt T. (1989). Differential effects of retinal target displacement, changing size and changing disparity in the control of anterior/posterior and lateral body sway. Exp Brain Res 78: 243–252. 2599035 10.1007/BF00228896

[27] Redfern M.S., Furman J.M., & Jacob R.G. (2007). Visually induced postural sway in anxiety disorders. J Anxiety Disord 21: 704–716. 10.1016/j.janxdis.2006.09.002 17045776 PMC1975822

[28] Simeonov P., & Hsiao H. (2001). Height, surface firmness, and visual reference effects on balance control. Injury Preven 7: i50–i53. 10.1136/ip.7.suppl_1.i50 PMC1765406 11565972

[29] Riccio G. E. (1993). Information in movement variability about the qualitative dynamics of posture and orientation In: Newell K. M., Corcos D. M., editors. Variability and motor control. Champaign, IL: Human Kinetics.

[30] Redfern M. S., Jennings J. R., Martin C., & Furman J. M. (2001). Attention influences sensory integration for postural control in older adults. Gait Pos 14: 211–216. 10.1016/s0966-6362(01)00144-8 11600324

[31] Ihlen E. A., & Vereijken B. (2010). Interaction-dominant dynamics in human cognition: Beyond 1/ƒ α fluctuation. J Exp Psychol: General 139: 436–463. 10.1037/a0019098 20677894

[32] Ihlen EA, Skjaeret N, Vereijken B (2013). The influence of center-of-mass movements on the variation in the structure of human postural sway. J Biomech 46: 484–490. 10.1016/j.jbiomech.2012.10.016 23149080

[33] Palatinus Z. Kelty-Stephen D. Kinsella-Shaw J. Carello C. Turvey M. (2014). Haptic perceptual intent in quiet standing affects multifractal sscaling of postural fluctuations. J Exp Psychol: Human Percep Perf, 40: 1808–1818. 10.1037/a0037247 24999615

[34] Thurner S., Mittermaier C., Hanel R., & Ehrenberger K. (2000). Scaling-violation phenomena and fractality in the human posture control systems. Physical Rev E, 62, 4018–4024. 10.1103/physreve.62.4018 11088924

[35] Turvey M. T., & Fonseca S. (2014). The medium of haptic perception: a tensegrity hypothesis. J Motor Behav 46: 143–187. 10.1080/00222895.2013.798252 24628057

[36] Kelty-Stephen D. G., Palatinus K., Saltzman E., & Dixon J. A. (2013) A tutorial on multifractality, cascades, and interactivity for empirical times series in ecological science. Ecol Psychol 25: 1–62

[37] Shimizu Y, Thurner S., &Ehrenberger K. (2002). Multifractal spectra as a measure of complexity in human posture. Fractals 10 103–116.

[38] Goldberger AL, Amaral LA, Hausdorff JM, Ivanov PC, Peng CK, Stanley HE (2002). Fractal dynamics in physiology: Alterations with disease and aging. Proc Nat Acad Sci 99(Suppl. 1):2466–2472. 11875196 10.1073/pnas.012579499 PMC128562

[39] Ihlen E.A. (2012). Introduction to multifractal detrended fluctuation analysis in Matlab, Frontiers Physiol 3: 141. 10.3389/fphys.2012.00141 PMC3366552 22675302

[40] Kantelhardt JW, Zschiegner SA, Koscielny-Bunde E, Havlin S, Bunde A, Stanley HE (2002) Multifractal detrended fluctuation analysis of nonstationary time series. Physica A Stat Mech Appl, 316, 87–114

[41] Beer T. (1997). Environmental oceanography. Boca Raton, FL: CRC Press.

[42] Yu Y., Yank J. R., Katsumata Y., Villard S., Kennedy R. S., & Stoffregen T. A. (2010). Visual vigilance performance and standing posture at sea. Aviat Space Environ Med 81: 375–382. 20377140 10.3357/asem.2638.2010

[43] Birren J. E., Woods A. M., & Williams M. V. (1980). Behavioral slowing with age: Causes, organization and consequences In Poon L. W. (Ed.), Aging in the 1980’s (pp. 293–308). Washington DC: American Psychological Association.

[44] Voelcker-Rehage C. (2008). Motor-skill learning in older adults—a review of studies on age-related differences. Euro Rev Aging Physical Activ 5: 5–16.

[45] Roller C. A., Cohen H. S., Kimball K. T., & Bloomberg J. J. (2002). Effects of normal aging on visuo-motor plasticity. Neurobio Aging 23: 117–123. 10.1016/s0197-4580(01)00264-0 11755026

[46] Jor’dan A. J., McCarten J. R., Rottunda S., Stoffregen T. A., Manor B., & Wade M. G. (2015). Dementia alters standing postural adaptation during a visual search task in older adult men. Neurosci Lett 593: 101–106. 10.1016/j.neulet.2015.03.014 25770830 PMC4509567

[47] Winter D. A., Prince F., Frank J. S., Powell C., & Zabjek K. F. (1996). Unified theory regarding A/P and M/L balance in quiet stance. J Neurophysiol 75: 2334–2343. 8793746 10.1152/jn.1996.75.6.2334

[48] Balasubramaniam R., Riley M. A., & Turvey M. T. (2000). Specificity of postural sway to the demands of a precision task. Gait Pos 11: 12–24. 10.1016/s0966-6362(99)00051-x 10664481

[49] Munafo J., Wade M. G., Stergiou N., & Stoffregen T. A. (2015). Subjective reports and postural performance among older adult passengers on a sea voyage. Ecol Psychol 27: 127–143.

[50] Chiari L, Rocchi L, Cappello A (2002). Stabilometric parameters are affected by anthropometry and foot placement. Clin Biomech 17: 666–677. 10.1016/s0268-0033(02)00107-9 12446163

[51] Kim J. W., Eom G. M., Kim C. S., Kim D. H., Lee J. H., Park B. K., & Hong J. (2010). Sex differences in the postural sway characteristics of young and elderly subjects during quiet natural standing. Geriat Geront Int 10: 191–198. 10.1111/j.1447-0594.2009.00582.x 20100287

[52] Sullivan E. V., Rose J., Rohlfing T., & Pfefferbaum A. (2009). Postural sway reduction in aging men and women: Relation to brain structure, cognitive status, and stabilizing factors. Neurbio Aging 30: 793–807. 10.1016/j.neurobiolaging.2007.08.021 PMC2684797 17920729

[53] Koslucher F. C., Munafo J., & Stoffregen Thomas A. (2016). Postural sway in men and women during nauseogenic motion of the illuminated environment. Exp Brain Res 234:2709–2720. 10.1007/s00221-016-4675-8 27236456

[54] van Wegen E. E. H., van Emmerik R. E. A., & Riccio G. E. (2002). Postural orientation: Age-related changes in variability and time-to-boundary. Hum Move Sci 21: 61–84. 10.1016/s0167-9457(02)00077-5 11983434

[55] Lajoie Y., & Gallagher S. P. (2004). Predicting falls within the elderly community: comparison of postural sway, reaction time, the Berg balance scale and the Activities-specific Balance Confidence (ABC) scale for comparing fallers and non-fallers. Arch Geront Geriat 38: 11–26. 10.1016/s0167-4943(03)00082-7 14599700

[56] Cruise Lines International Association, Inc. (2010). 2010 CLIA Cruise Market Overview Fort Lauderdale FL: Cruise Lines International Association, Inc.

